# New Star-Shaped Polyether-Pentols (PEPOs) for Fabrication of Crosslinked Polyurethanes—Synthesis and Characterization

**DOI:** 10.3390/polym13132150

**Published:** 2021-06-29

**Authors:** Justyna Jurek-Suliga, Zbigniew Grobelny, Sylwia Golba, Hubert Okła, Katarzyna Bednarczyk

**Affiliations:** 1 Institute of Engineering Materials, University of Silesia, 41-500 Chorzow, Poland; sylwia.golba@us.edu.pl (S.G.); hubert.okla@us.edu.pl (H.O.); 2 Institute of Chemistry, University of Silesia, 40-007 Katowice, Poland; zbigniew.grobelny@us.edu.pl (Z.G.); k.bednarczyk@us.edu.pl (K.B.)

**Keywords:** polymer synthesis, molecular engineering, ROP, oxiranes anionic polymerization, potassium hydroxyalkoxides, macropentols

## Abstract

Polyether-pentols (PEPOs) were synthesized from glycidyl ethers and butylene oxide with the application of tripotassium salts of 2,2,6,6-tetrakis(hydroxymethyl)cyclohexanol (HMCH) activated 18C6 for ring-opening polymerization (ROP). The construction of the applied initiator system reflects the ability of crown ether to influence the degree of ion-pair separation with an increased activating effect. As a result formation of bi- or trimodal polymers was observed with molar masses in the range of (*M*_n_ = 1200–6000). The observed multi-fraction composition is prescribed to the formation of ionic aggregates with different reactivities during polymerization. The mechanism of the studied processes is discussed. The obtained PEPOs served for a crosslinked PUR synthesis, for which the hydrogen bond index for coupling of hard segments was calculated. Additionally, the range of phase separation was calculated that was higher for PUR-containing aromatic rings as the substituent.

## 1. Introduction

Polyether-polyols (PEPOs) belonging to foaming polymer are based on the polyoxyalkylene glycols (e.g., dipropylene glycol), the higher alkyleneoxy polyols (e.g., polypropylene glycol), and also ethylene or propylene oxide (EO or PO respectively) adducts of polyfunctional alcohols and polyfunctional amines such as pentaerythritol, sorbitol, 2,2,6,6.-tetrakis(hydroxymethyl)cyclohexanol, triethanolamine, and polyoxybutylene glycols [1]. PEPOs are important macromonomers utilized for the synthesis of polyurethane elastomers and crosslinked PUR foams [1,2,3,4,5,6]. In this synthesis, the alkali-catalyzed anionic polymerization takes place, where different di- or polyfunctional starting alcohols (initiators) with epoxides (ethylene and/or propylene oxide) react at high temperature (>100 °C) and pressure [7]. To obtain a well-characterized product, one shall control the selectivity of propagating reactive species. This is possible by utilization methods such as controlled or living polymerization [8]. From a mechanism point of view, the main synthesis routes involve ring-opening (ROP) of epoxides by either coordination or ionic mechanism.

The anionic polymerization enables the control of the features of the final product, such as molar mass or dispersity [8]. In the polyethers synthesis, several crucial factors must be taken into consideration, such as ring strain and polymerization enthalpy, as they directly influence epoxide reactivity and polymerization kinetics. It was also proven that electronic and steric factors associated with the nature of the ring substituent, as well as reaction conditions (temperature and solvent), could play an important role [9].

Recently [10], we used for initiation dipotassium salts of different glycols (e.g., di- or tripropylene ones, activated 18-crown-6 (18C6)). It allowed preparing bimodal PPOs containing fraction with two terminal OK groups (*M*_n_~10,000) and fraction with allyloxy and OK terminal groups (*M*_n_~30,000). Both kinds of terminal groups can be easily converted to OH ones [1]. The structure of the polyols influences the property of the polyurethanes. Hence this can be a factor controlled by the selection of the length and composition of the polyether chains and the functionality (f) of the monomers [11]. In this way, PEPOs create a group of polymers whose members are characterized by a variety of properties. If flexibility is urged, polyols are synthesized with low functionality initiators such as dipropylene glycol (f = 2) or glycerine (f = 3). On the other side, if rigidity is required, then high functionality initiators such as sucrose (f = 8), sorbitol (f = 6), toluenediamine (f = 4) are used. Tetra- and higher functional PEPOs are usually obtained in the reaction of an alkylene oxide (ethylene oxide, propylene oxide, butylene oxide) with a polyol possessing four or more hydroxyl groups [8].

To tailor mutual interaction in the structure of the initiator system, we previously applied cyclic oligo (potassium glycidoxide) with three or six OK groups activated 18C6 as macroinitiators for the polymerization of PO, BO, and styrene oxide (SO) [12]. Star-shaped polyether-polyols with *M*_n_ = 1200–8000 were prepared this way. These polymers can be used as substrates for the synthesis of new polyurethanes with improved mechanical properties.

The aim of the present work was the application of new initiating systems with a tailored strength of ionic pair generations for the preparation of new star-shaped polyether polyols (PEPOs) based on the various epoxy-ring monomers. The initiating system is constructed with potassium hydroxyalkoxides synthesized in the reaction of KH activated 18C6 with 2,2,6,6-tetrakis(hydroxymethyl)cyclohexanol (THMC). In the core initiator structure (THMC), the central six-membered ring adopts a slightly twisted chair conformation. For this molecule, the crystal packing is stabilized by mutual intermolecular O–H∙∙∙O interactions [13,14]. THMC was reported to be useful as a functional hydroxyl monomer for the synthesis of crosslinked polyorthocarbonates [15], ladder-type polyspiroacetals [16,17], and also polyurethane rigid foams prepared from soy-based polyol derived from a reaction with cyclohexanol [18].

**Figure 1:**
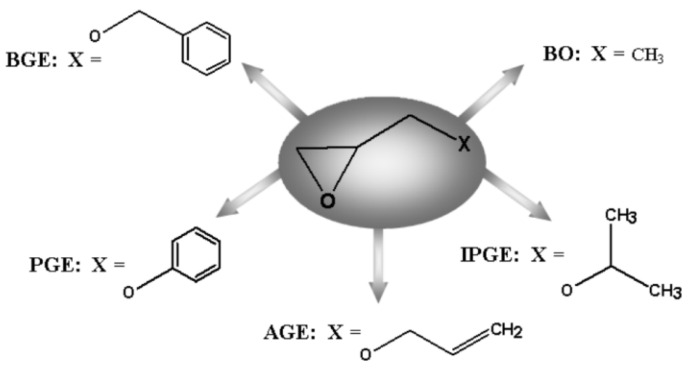
Structure of monomers utilized for the synthesis of PEPOs.

Application of bio-polyols is currently a widely investigated scientific path, where polyols derived from natural sources are subjected to epoxidation and further oxirane rings opening reaction [19,20] to improve heat-insulating properties of foams. Except for the possibility of building a star shape structure for growing polyether-polyols, the presence of THMC can induce reduced amounts of toxic fumes on burning the final polyurethane composition, as stated by Bak [21]. BO, isopropyl glycidyl ether (IPGE), allyl glycidyl ether (AGE), phenyl glycidyl ether (PGE), and benzyl glycidyl ether (BGE) were used as monomers for the synthesis of polymers (Figure 1). All syntheses were performed in mild conditions, i.e., in tetrahydrofuran (THF) solution at room temperature. Characterization of polymers was performed by several techniques such as ^13^C NMR, MALDI-TOF, SEC, and FTIR techniques.

## 2. Experimental Section

### 2.1. Materials

Monomers, i.e., 1,2-butylene oxide, isopropyl glycidyl ether, allyl glycidyl ether, phenyl glycidyl ether and, benzyl glycidyl ether (all from Aldrich/Poland) were purified in a two-step procedure with drying over CaH_2_ at first and distillation under reduced pressure (11 mmHg) at an appropriate temperature in the second step—at 336 K (63 °C) for BO, 414 K (131 °C) for isopropyl glycidyl ether (IPGE), 427 K (154 °C) for allyl glycidyl ether (AGE), 518 K (245 °C) phenyl glycidyl ether (PGE) and 344 K (71 °C) for benzyl glycidyl ether (BGE). Commercially available anhydrous tetrahydrofuran (THF) (Acros Organics, Poznan, Poland, water content max 50 ppm) was additionally kept over CaH_2_ and distilled at 339 K (66 °C) to remove traces of impurities that could induce solvent polymerization [22,23]. Potassium hydride (KH) was purified according to the procedure [24] where a dispersion of KH in mineral oil (35 wt%, Sigma Aldrich, Poznan, Poland) was mixed with *n*-pentane under sealing dry argon atmosphere and then decanted. The triple repetition of this step followed by triple washing with dry THF resulted in a satisfactory purity level. Finally, THF was evaporated in a vacuum. The presence of KH in the system was determined by a standard gas law calculation of the amount of hydrogen liberated after treatment with 2-butanol (1.0 H_2_ = 1.0 KH). The resulting solution was titrated to a phenolphthalein endpoint. Very little excess (<1%) of total base over hydride base (from gas evolution) indicated small hydrolysis of the original KH sample. Coronand 18C6 (1,4,7,10,13,16-hexaoxacyclooctadecane) (Merck, Warsaw, Poland) and 2,2,6,6-tetrakis(hydroxymethyl)-cyclohexanol (THMC, Aldrich, Warsaw, Poland) were used for synthesis without further purification.

### 2.2. Polymerization

Tripotassium salts of 2,2,6,6-tetrakis(hydroxymethyl)cyclohexanol (3K-THMC) activated 18C6 were prepared in the reaction of pure THMC with an appropriate amount (calculated) of KH in THF at 20 °C. The initial concentrations of the monomer were 3.0 mol/dm^3^, and the initial concentration of the initiator was 0.02 mol/dm^3^. For example, in the synthesis of 3K-THMC activated 18C6 potassium hydride (0.047 g, 1.2 mmol), THF (4.0 cm^3^) and 18C6 (0.32 g, 1.2 mmol) were introduced into the reactor. Then, THMC (0.088 g, 0.4 mmol) was dissolved in boiling THF (10.0 cm^3^), and the solution obtained was dropped to the system at 20 °C. After mixing for 1.5 h hydrogen (26.8 cm^3^) was evolved. Finally, 1,2-butylene oxide (1,2-epoxybutane) (5.22 cm^3^, 4.32 g, 60 mmol) was added and, the reaction mixture was stirred for several days. After complete conversion of the monomer, the reaction mixture was treated with H_2_O or HCl/H_2_O system (0.1 mol/dm^3^, 70 cm^3^) and transferred to the separator containing chloroform (70 cm^3^). After shaking for 5 min, two layers were formed, i.e., an inferior polyether layer and a superior layer containing water and potassium salt. These layers were separated and, the superior layer was removed. After three items of washing with distilled water, polyether was obtained by evaporating chloroform and water. In the studied systems, the final conversion was 97–99% after 1–3 weeks. Heterogeneity was observed in the systems containing 3K-THMC salt.

### 2.3. Measurements

100 MHz ^13^C nuclear magnetic resonance (NMR) spectra were recorded in CDCl_3_ at 25 °C on a BrukerAvance 400 pulsed spectrometer (Bruker, Billerica, MA, USA) equipped with a 5-mm broad-band probe and applying the Waltz16 decoupling sequence. Chemical shifts were referenced to an internal standard (TMS—tetramethylsilanem). To reveal microstructural details of the polymer main chain high-quality spectrum must be recorded, with 3000 scans being a satisfactory amount, however, to observe the signals of the polymer chain ends, more than 10,000 scans were necessary.

Molar masses and dispersity (d) of polymers were determined by means of size exclusion chromatography (SEC) on a Shimadzu Prominance UFLC instrument (Shimadzu, Kyoto, Japan) at 40 °C on a Shodex 300 mm × 8 mm OHpac column using tetrahydrofuran as a solvent. PS were used as calibration standards.

Matrix-assisted laser desorption/ionization-time of flight (MALDI-TOF) spectra were recorded on a Shimadzu AXIMA Performance instrument with dithranol used as a matrix.

Infrared (IR) spectra of samples were recorded on a Shimadzu IR Prestige spectrometer with an attenuated total reflectance (ATR) accessory. The diamond ATR crystal was purified prior to each measurement with isopropanol. Data were analyzed using the LabSolutions program. Each sample was scanned at a resolution of 2 cm^−1^.

Differential Scanning Calorimetry (DSC) was performed using the Mettler Toledo apparatus. Samples were heated, cooled, and reheated with a speed of 10 °C/min, in the temperature range from −100 to 120 °C. The DSC curves taken for the analysis were obtained from the second run. Temperature calibration was performed with indium (melting temperature = 156.6 °C), heat of fusion (∆*H*_f_ = 28.5 J/g).

A thermal gravimetric analyzer (TA-DSC 2010 (TA Instruments, New Castle, DE, USA) was employed to measure the thermal degradation of PUR samples. It was performed under a nitrogen atmosphere (50 mL/min) by increasing the temperature in a range of 20 to 700 °C at a heating rate of 10 °C/min and cooling rate of 20 °C/min in closed aluminum vessels. Measurements were carried out for samples weighing 7.5–10.0 mg, measurements and analyzes were processed using Thermal Solution Software (Cleveland, OH, USA).

The PURs samples were imaged using a high-resolution X-ray scanner (v|tome|x s, GE Sensing & Inspection Technologies, Hurth, Germany; Phoenix|X-ray, Wunstorf, Germany). The difference in the degree of X-ray absorption, which depends on the material density, allows the visualization of the internal microstructure of samples in micron or submicron resolution. The PURs were placed in a suitable rack and scanned at 80 kV and 150 μA. For each sample, 1000 projections were recorded at a total scanning time of 20 min. The X-rays, after passing through the sample, were transformed into visible radiation using a YAG: Ce scintillator. Consequently, the images were recorded with a resolution of 2024 × 2024 pixels. The distance of the sample from the matrix was selected to obtain the maximum image resolution. The parameters determined in this way allowed to register an image with optimal contrast and a resolution of 10 µm. The acquisition of scans was carried out in 8-bit grayscale in order to most accurately identify changes in the density of the analyzed PURs. Datos 2.0 software (GE Sensing & Inspection Technologies, Hurth, Germany; Phoenix|X-ray, Wunstorf, Germany) and VGStudio MAX 2.1 (Volume Graphics, GmbH., Heidelberg, Germany) were used, respectively, for acquisition and image reconstruction.

## 3. Results and Discussion

### 3.1. Synthesis of Star-Shaped Polyether-Polyols (PEPOs) by Use of Potassium Salts of 2,2,6,6-Tetrakis(hydroxymethyl)cyclohexanol (THMC) Activated 18C6

**Scheme 1:**
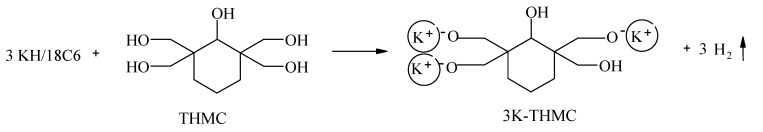
Synthesis of tripotassium salt of 2,2,6,6-tetrakis(hydroxymethyl)cyclohexanol (a circle denotes 18C6).

To start the polymerization, new initiator systems were obtained, where tripotassium salt of THMC was prepared in the reaction with KH suspension in THF (Scheme 1).

Although we observed that solubility of the pristine pentol in THF is low (close to 0.04 mol/dm^3^) but the presence of crown compound 18C6 allowed to obtain its potassium salt (denoted as 3K-THMC, which was used as an initiator of chosen oxiranes polymerization (Scheme 1). Crown ethers form stable complexes with metal cations if the size of the crown ether cavity matches that of the cation [25]. This interaction results in an increase in the degree of ion-pair separation. In the anionic ROP of propylene oxide and other oxiranes, complexations of the cation cause a marked activating effect, i.e., positive effect on reaction rate and yield of the product [26,27]. In the presented study, five oxiranes were used as monomers to synthesize star-shaped polymers, which were 1,2-butylene oxide (BO), isopropyl glycidyl ether (IPGE), allyl glycidyl ether (AGE), phenyl glycidyl ether (PGE), and benzyl glycidyl ether (BGE). Data concerning molar masses and dispersity of obtained PEPOs polymers are presented in Table 1.

**Figure 2:**
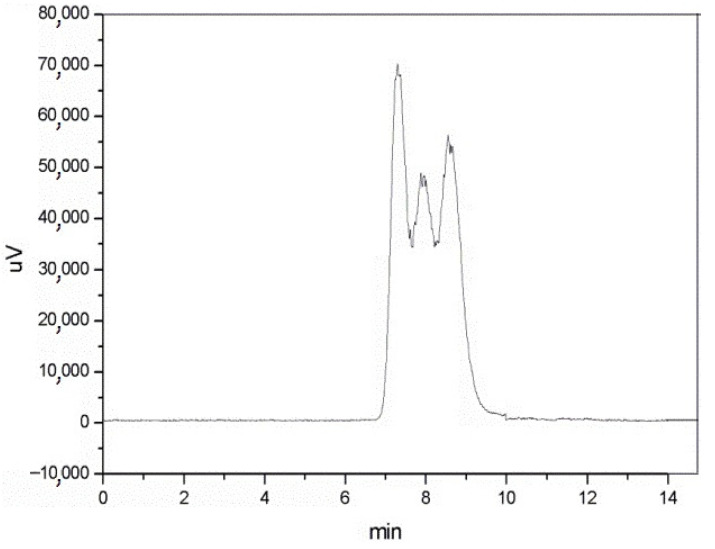
SEC chromatogram of PIPGE (2).

Closer examination of the obtained results indicates that final polyethers are either unimodal (1), bimodal (4), or even trimodal (2, 3, 5). Figure 2 shows, as an example, the SEC chromatogram of PIPGE-pentol (2).

The observed multi-fraction composition urges some comments. It is a generally accepted view that in the polymerization process, bimodality exclusively occurs when there are two types of species propagating with different rate constants, and these species do not exchange quickly enough [28]. In the studied systems, macromolecules contain terminal, i.e., starting and end alkoxide and hydroxyl groups, which interact mutually and form ionic aggregates with different reactivities. This phenomenon is probably responsible for the formation of two or three polymer fractions.

**Figure 3:**
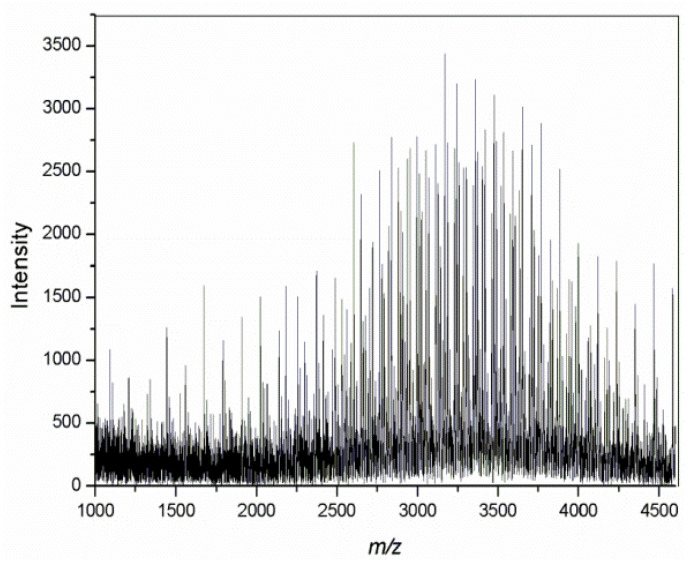
MALDI-TOF spectrum of PIPGE (2).

**Figure 4:**
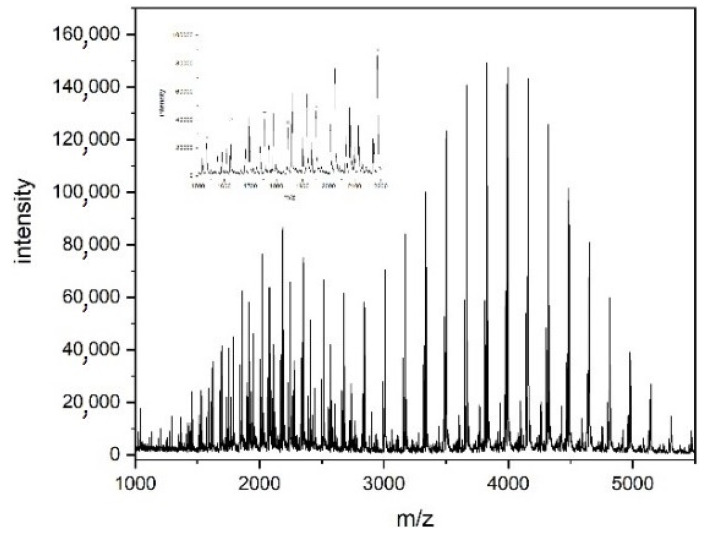
MALDI-TOF spectrum of PBGE (5).

In general, spectra MALDI-TOF of the obtained polymers (1–5) are well resolved and present a broad range of molecular masses. Figure 3 and Figure 4 show, as an example, spectra of PIPGE (2) and PBGE (5), respectively.

MALDI-TOF spectrum of polymer (2) at *m*/*z* 1000 to 4500 reveals several series of signals. The signals of the main series, for instance at *m*/*z* 2024.1, 2605.0, and 3767.1 represent macromolecules possessing a central part derived from the initiator as well as five PIPGE arms with four OH and one ONa end groups. These macromolecules contain 15, 20, and 30 mers of isopropyl glycidyl ether, respectively, and form adducts with one potassium ion (*M*_calc_ = 2023.8, 2604.6 and 3766.2, respectively). The second series of the signals, for example at *m*/*z* 2471.5, 3171.5, and 3751.0 represents macromolecules with the same structure containing 19, 24, and 30 mers of isopropyl glycidyl ether, respectively, but forming adducts with one sodium ion (*M*_calc_ = 2472.3, 3169.3 and 3750.1, respectively). An additional series of signals represent macromolecules with various numbers of potassium and sodium ions.

**Scheme 2:**
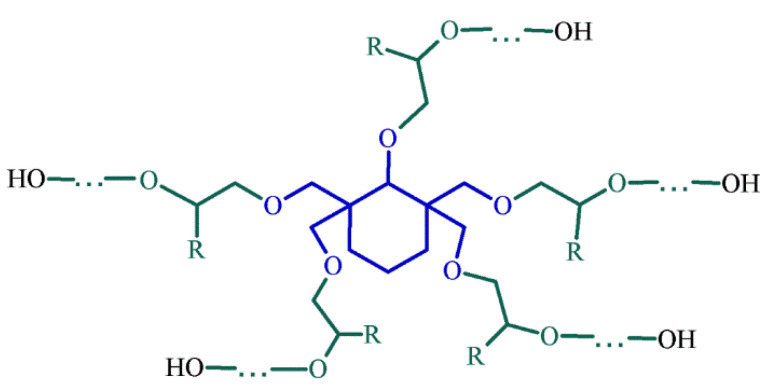
Polyether-pentols (PEPOs) formed in the polymerization of oxiranes initiated with potassium salts of 2,2,6,6-tetrakis(hydroxymethyl)cyclohexanol. R: CH_3_CH_2_ (for BO), (CH_3_)_2_CHOCH_2_ (for IPGE), CH_2_ = CHCH_2_OCH_2_ (for AGE), PhOCH_2_ (for PGE), PhCH_2_OCH_2_ (for BGE).

MALDI-TOF spectrum of polymer (5) (Figure 4) at *m*/*z* from 1800 to 5500 shows several series of signals. The signals of the main series, for example, at *m*/*z* 1530.8, 2185.8, and 3827.6, (marked with a circle) represent macromolecules containing central parts derived from the initiator as well as five PBGE arms with one, two ONa, and two OK end groups. These macromolecules contain 7, 11, and 21 mers of BGE, respectively, and form adducts with one potassium ion (M_calc_ = 1529.0, 2185.8, and 3827.8, respectively). The second series is also observed at *m*/*z* from 800 to 5500. For example, the peaks at *m*/*z* 1843.2, 2338.7, and 2991.0 (marked with a square) represent macromolecules with 9, 12 and 16 mers of benzyl glycidyl ether, respectively (M_calc_ = 1845.3, 2337.9, and 2994.7, respectively). These macromolecules form adducts with three sodium ions and two potassium ions. The next three series of signals at *m*/*z* 800 to 3500 belong to macromolecules with one OH group and three ONa groups which form adducts with one sodium or one potassium ions, as well as macromolecules with three OH groups and one ONa group which form adducts with one sodium ion, respectively. The first one involving signals, for example at *m*/*z* 1622.9, 1951.1 and 2443.6 (marked with an empty circle) represent macromolecules containing 8, 10, and 13 mers of benzyl glycidyl ether, respectively (M_calc_ = 1622.7, 1951.3, and 2443.4, respectively). The second series of peaks, for example, at m/z 1753.4, 2408.5, and 2736.1 (marked with an empty square) presents macromolecules with 9, 13, and 15 mers of BGE, respectively (M_calc_ = 1757.2, 2414.0, and 2742.4, respectively). The third series of signals, for example at *m*/*z* 2064.4, 2228.9 and 2720.3 represents macromolecules with 11, 12 and 15 mers of BGE, respectively (M_calc_ = 2071.3, 2235.7, and 2728.3, respectively). The last series of peaks is observed at *m*/*z* from 3100 to 5500. For example, peaks at m/z 3437.4, 4095.6, and 5079.5 represent macromolecules with 19, 23, and 29 mers of BGE, respectively, containing three OH groups and two OK groups and forming an adduct with one sodium ion (M_calc_ = 3439.3, 4096.1, and 5081.3, respectively). However, we observed that the use of H_2_O/HCl as a quenching agent results in star-shaped macromolecules with five polyether-arms with OH end groups (Scheme 2).

**Figure 5:**
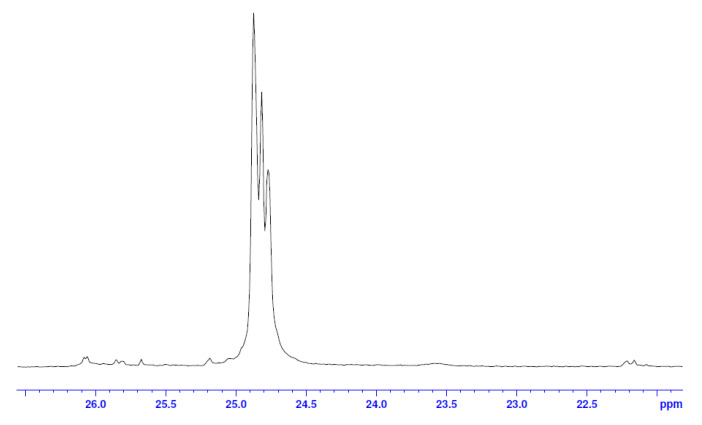
^13^C NMR spectrum of PBO (1).

Incorporation of the initiator cyclohexane ring into the polyether-pentols (PEPOs) cannot be simply confirmed by NMR due to limited solubility of the initiator in deuterated chloroform used to record the spectra of the polymers, which makes direct tracking of the initiator difficult. However, a comparison of the initiator and the polymer ^13^C NMR spectra recorded in deuterated DMSO confirmed the presence of the three methylene carbons from the cyclohexane ring in the polymer molecules, for example, at 24.87 ppm, 24.82, and at 24.77 ppm (C3-C5) in the case of polyether-pentol with poly(1,2-butylene oxide) arms (Figure 5).

**Figure 6:**
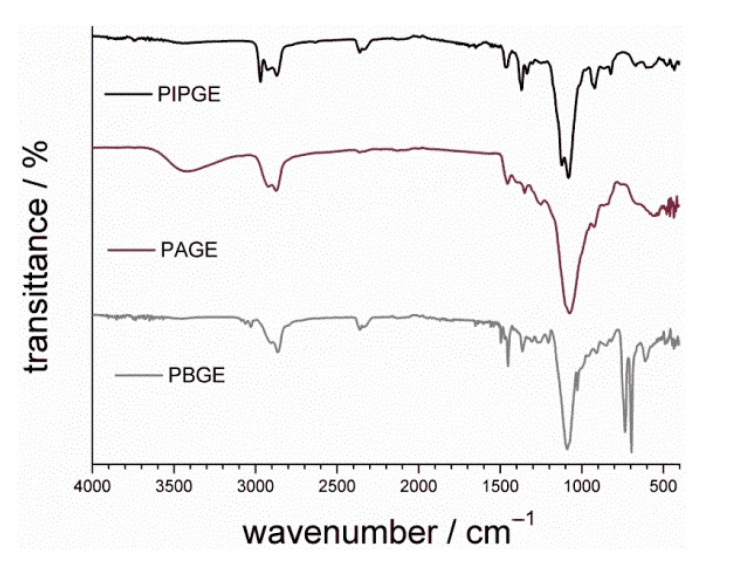
Exemplary FTIR spectra of star-shaped polyether-pentols (PEPOs) with arms built with isopropyl glycidyl ether (PIPGE (2)), allyl glycidyl ether (PAGE (3)) and benzyl glycidyl ether (PBGE (5)).

Five obtained star-shaped polyethers, FTIR ATR spectra were also recorded to confirm their structures (Figure 6). Bands at 3000–2850 cm^−1^ reflect alkane-like fragments (–CH_2_–) of the main chains together with bending/scissoring vibration of C–H in the range 1470–1450 cm^−1^ and rocking ones of C-H at 1370–1350 cm^−1^. The presence of an aromatic phenyl ring is manifested with =C–H stretching bands located in the range above 3000 cm^−1^ (3100–3000 cm^−1^) (for PPGE (4) and PBGE (5)). The absorption within the range of 1600–1585 cm^−1^ and 1500–1400 cm^−1^ is caused by stretching bands of –C=C– in aromatic rings. Furthermore, a clear indication of the aromatic character of the substituent is shown by 800–675 cm^−1^ out-of-plane vibration bands. Bands at 770–730 cm^−1^ and 710–690 cm^−1^ indicate monosubstitution of the aromatic ring (such as phenyl). The band at appr. 1640 cm^−1^ (for PAGE (3)) results from stretching of the alkenyl –C=C– couple together with low-intensity bands at 1430–1310 cm^−1^ from vinyl C–H in-plane bending. For synthesized polymers, bands at 3492 cm^−1^ represent stretching vibration of associated –O–H. The absorption peak at 1318 cm^−1^ is attributed to O–H bending vibration. Complementary C–O stretching band is visible in the range 1260–1050 cm^−1^ with a 1060 cm^−1^ stretching band of C–O for primary alcohol. It is overlaid with ether C–O–C band located at 1095 cm^−1^ which is characteristic of alkyl-substituted ether.

### 3.2. Preparation and Characterization of Crosslinked Polyurethanes

Synthesized star-shaped polyethers, after purification, i.e., removing of alkali metal cations and water, were used for the fabrication of polyurethanes. The reaction was performed with polydiisocyanate (PMDI), resulting in crosslinked polyurethanes. As polyol components, three PEPOs were chosen, which were PBGE (5) for PUR1, PBO (1) for PUR2, and PIPGE (2) for PUR3. During polyurethane production, the polyols and PMDI were mixed with a mechanical stirrer at 1000 rpm for 20 min with the NCO/OH ratio equal to 5.0. The reaction (one-step polycondensation) took place at room temperature, and a cooling system was applied to keep the temperature of the system at 40 °C. The relative humidity was kept below 30%. One of the most important tools used to describe the PUR chemical structure and phase separation process is infrared spectroscopy (IR) [29]. A qualitative description of the chemical structure of crosslinked PURs makes it easy to compare the characteristic absorption bands of the materials.

**Figure 7:**
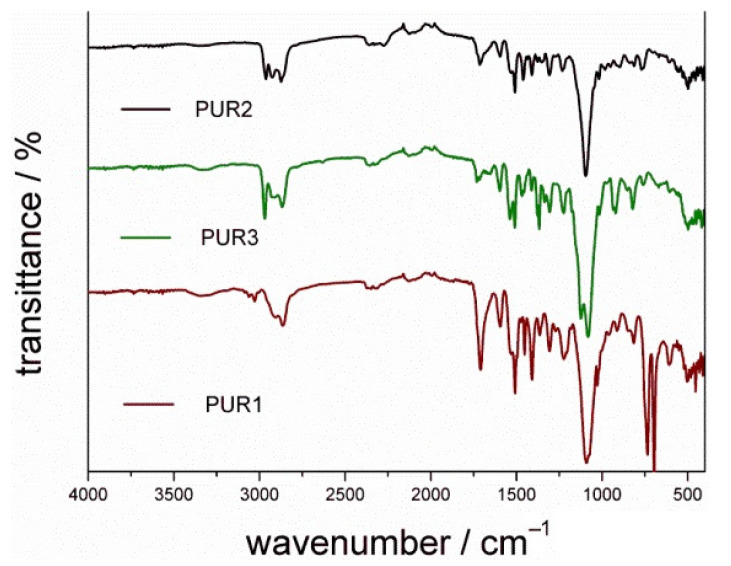
FTIR spectra of polyurethane based on and benzyl glycidyl ether PBGE (PUR1)/1,2-butylene oxide PBO (PUR2)/isopropyl glycidyl ether PIPGE (PUR3) and PMDI.

FTIR spectra of cross-linked polyurethane (Figure 7) reveal peaks at 2855 and 2930 cm^−1^ that reflect symmetric and nonsymmetric vibration of C–H and CH_2_ with the presence of ether moiety confirmed by the band at 1100 cm^−1^. Peaks at 3340 cm^−1^ reflect stretching vibration of N–H II-order groups of urethane bond. The wide peak at 3550–3100 cm^−1^ indicates the presence of hydrogen bonding between chains. Stretching bands for free NH, not involved in hydrogen bonding, are located at 3420 cm^−1^. It is overlapped with a shoulder band at 3250 cm^−1^ which reflects stretching bands of hydrogen-bonding for NH–O– couple below *T*_g_ of soft segments. Structural analysis has shown that the polyurethanes have segregated structures with hard and soft domains separated into two phases [30]. The driving force of the interaction is the possibility of forming weak non-covalent bonds between the molecular segments formed by the rigid linking the NH group with carbonyl groups (–C=O), which can be investigated based on the carbonyl absorption region (from 1740 to 1660 cm^−1^) [31].

**Figure 8:**
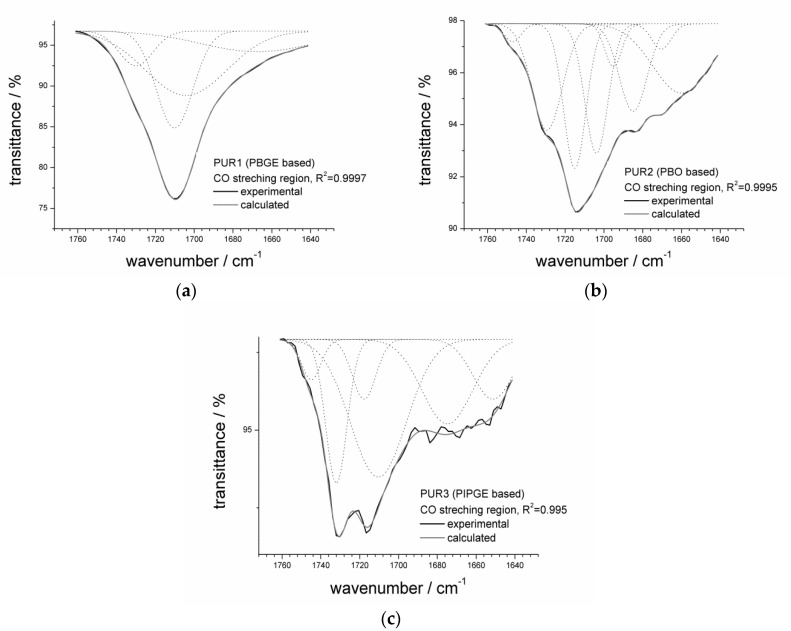
The Fourier deconvoluted spectrum for the carbonyl stretching region of the investigated PURs; (**a**) PUR1, (**b**) PUR2, (**c**) PUR3.

The Fourier deconvoluted spectrum for the carbonyl stretching region of the PURs sample is shown in Figure 8. In the range, 1745–1720 cm^−1^, the band of the free carbonyl group is observed, which is present in the mixed soft segment domain or at the interface. The bands in region 1720 to 1710 cm^−1^ correspond to the associated hydrogen bonding of a carbonyl group in the disordered phase, while the ordered phase bonding occurs in the region 1709 to 1695 cm^−1^. They represent the self-association of N–H and O=C in the interior of the hard domain, and the exact position depends on the strength of the hydrogen bonding. To characterize qualitative samples, a factor called hydrogen bond index for coupling of hard segments (R) was calculated. It is derived as a ratio between the peaks’ area for the bonded carbonyl part (in both ordered (CO bound or) and non-ordered phase (CO bound non-or)) versus the peak area for free carbonyl moiety (CO free) (Table 2). Using the obtained R-value, two other factors were calculated, namely the range of phase separation (RPS) and the range of hard phase dispersion (RPD) [32,33]. RPS describes the content of hard segments bonded to each other, while RPD refers to the content of hard segments non-bonded to other segments. While studying the calculated parameters, one can conclude that for PUR based on one of alkyl-based PEPOs (PBO (PUR2) and PIPGE (PUR3), the presence of a more bulky isopropyl-substituent (PIPGE) diminishes the tendency to phase separation, most probably owing to its size. Moreover, for alkyl-based PEPOs, the chains are less prone to phase separation in comparison to aromatic-based ones (PBGE (PUR1)). This reflects the tendency of interaction between neighboring aromatic phenyl rings. In this way in the range of the hard phase, dispersion is lower.

**Figure 9:**
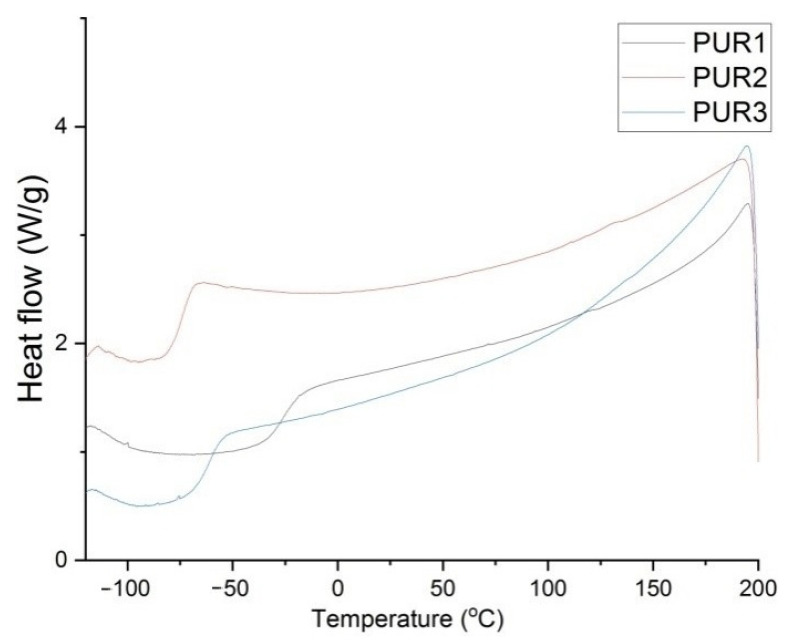
Thermograms for polyurethane based on benzyl glycidyl ether PBGE (PUR1)/1,2-butylene oxide PBO (PUR2)/isopropyl glycidyl ether PIPGE (PUR3) and PMDI.

For the prospective application of the obtained material, PUR thermal resistance is important. One of the techniques for analyzing the thermal behavior of polyurethanes is differential scanning calorimetry (DSC). It enables a description of the PUR structure by determining the temperature and effects associated with physical changes in these materials [34,35,36]. Figure 9 shows the thermograms of the obtained polyurethanes.

The highest glass temperature was noted for the PUR1 sample (−19.32 °C). This increase in comparison to PUR2 (−68.21 °C) and PUR3 (−54.93 °C) is due to the limitation of mobility of the flexible segments. For PUR1 flexible segments, higher energy is required to move the chains. This suggests the presence of some additional interaction of intra/intermolecular nature. We think that it could be an effect of hydrogen-bonding interaction as well as π–π interaction of aromatic rings in isocyanate segments. For star-shaped PUR1 based on benzyl glycidyl ether (PBGE), the structure favors hydrogen-bonds formation, which in turn enables mutual π–π interaction. In this case, the possibility of close interaction between aromatic rings is higher, and as a result, higher T_g_ is observed for PUR1. For PUR2 and PUR3, aromatic rings, the interaction seems to be less plausible as a linear structure imparts more flexibility for the segments of the macromolecules.

**Figure 10:**
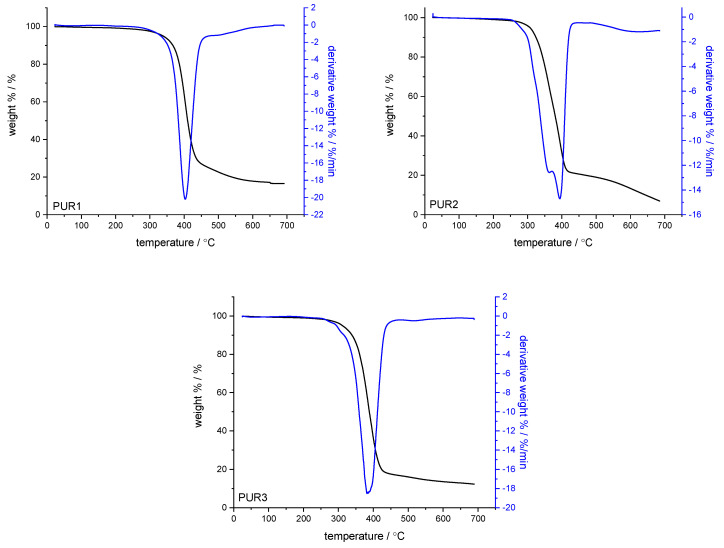
TG and DTG curve for cross-linked polyurethane based on benzyl glycidyl ether PBGE (PUR1)/1,2-butylene oxide PBO (PUR2)/isopropyl glycidyl ether PIPGE (PUR3) and PMDI.

Another technique for analyzing the thermal behavior of polyurethanes is TGA analysis. Figure 10 shows the TGA and DTG curves of the PURs foams. Their TGA parameters are shown in Table 3.

From the course of the sample mass curves against temperature, a 5% loss in mass of the sample (the first degree of irreversible decomposition), a 15% loss in mass of the sample, thermal responses of the sample, and mass residues were determined.

As shown by the TGA curves, the thermal degradation of the foams occurred at temperatures of 300 °C to 500 °C. 5% weight loss of the sample occurred at the temperature of 306.4 °C (PUR2), 311.3 °C (PUR3), and 338.3 °C (PUR1). The sequence for 15% weight loss is the same with the corresponding temperatures as high as 335.5 °C (PUR2), 352.1 °C (PUR3), and 378.6 °C (PUR1).

The first, low-intensity thermal response of the samples occurred at the temperature of 277.9 °C (PUR2) and 309.5 °C (PUR3), which is accompanied by a minor loss in the sample mass due to the removal of low molecular weight volatile components of the samples (3.41% and 6.35% respectively) during the early stage of thermal degradation. The step is not visible for the PUR1 sample.

Subsequent thermal responses related to PUR main chains decomposition occur as a two-stage process for PUR2 samples and a complex one-stage process for PUR3 and PUR1 samples. In the case of the PUR2 sample, the 2nd thermal response is visible at 363.5 °C with 39.22% weight loss, followed by 3rd step at 393.9 °C with 36.10% of weight loss. A similar two-stage thermal decomposition was reported for PURs [37,38].

Both stages are almost equivalent in terms of weight loss indicating the presence of two main types of bonds in the macrochains. They are a reflection of the decomposition of urethane linking within the PUR hard segments (2nd stage), leading to depolymerization of MDI-based diisocyanate. An increase in temperature allows for the further decomposition of most urethane and urea or ester bonds caused by the degradation of soft segments of the polyols (3rd stage) [39].

For one-step response systems, the 2nd thermal response is visible at 381.9 °C with 75.70% of weight loss for PUR3 samples and at 403.0 °C with 74.57% of weight loss for PUR1 samples. The more symmetric shape of the DTG curve implies a more uniform structure of the macrochains with one dominant type of bond. The residue after testing was 6.95%, 12.34%, and 16.59% of the original sample weight for the series PUR2, PUR3, and PUR1, respectively. The heat resistance of the investigated PURs is similar to the reported PURs based on the comparison of the main stage of degradation (2nd) [38,39]. In the studied series, sample PUR1 is the most thermally stable, which is the outcome of the presence of the aromatic ring in the primary diole structure. The presence of the isopropyl substituent (PUR3 sample) imposes slightly higher thermal stability over methylone (PUR2 sample) due to steric impact.

**Figure 11:**
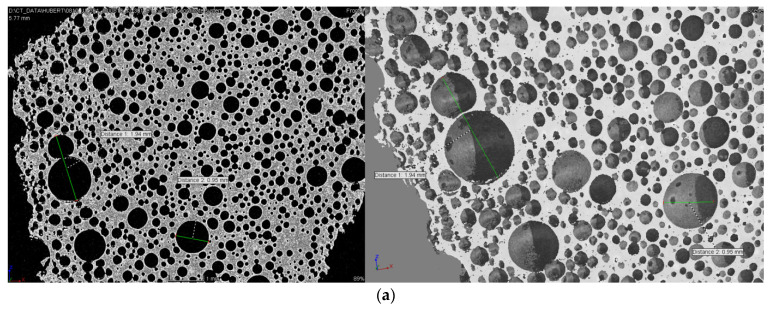

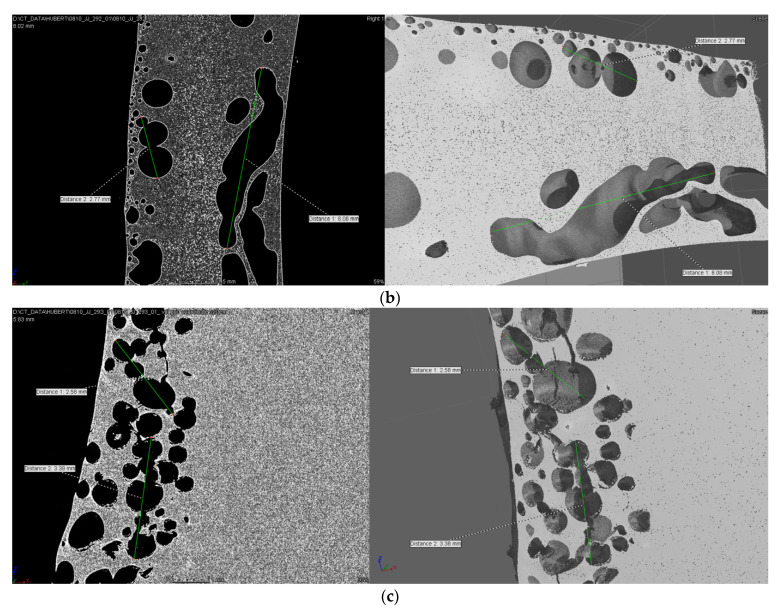
The PUR foam microstructure visualized using X-ray computed microtomography. Gray-scale reconstructed 2D and 3D images (**a**) PUR1, (**b**) PUR2, (**c**) PUR3.

To visualize the microstructure of the studied polyurethane foams, X-ray computed microtomography was performed [40,41,42]. A qualitative examination of the microtomographic imaging revealed differences in the shape and arrangement of pores in study foams. In the case of PUR1, the pores were distributed throughout the cross-section, their shape was regular and the maximum size did not exceed 1.94 mm. However, in the case of PUR2, and PUR 3, the free spaces were located mainly near the surface and their maximum size did not exceed 8.08 mm. The pores in PUR2 had a regular wall surface, while in PUR 3, the pores were connected by microcracks (Figure 11).

## 4. Conclusions

Tripotassium salts of 2,2,6,6-tetrakis(hydroxymethyl)cyclohexanol (THMC) activated 18-crown-6 are effective initiators of the polymerization of several monosubstituted oxiranes, i.e., 1,2-butylene oxide and four various glycidyl ethers. The processes were carried out in mild conditions, i.e., in THF solution at room temperature. The main characteristic features of the studied processes are:

- Application of potassium salt of THMC allowed us to synthesize polyether-pentols with the formation of bi- or trimodal polymers with molar masses in the range *M*_n_ = 1200–6000.
- The applied initiator system reflects the ability of crown ether to influence the degree of ion-pair separation, and hence the observed multi-fraction composition is prescribed to the formation of ionic aggregates with different reactivities during polymerization.
- MALDI-TOF and FITR spectra confirmed a star-shaped structure and composition of macromolecules with five polyether-arms with OH end groups.
- Polyether-polyols prepared in this work can be used for the synthesis of new polyurethanes.
- Analysis of the carbonyl stretching region in FTIR spectra of crosslinked polyurethanes revealed that in the presence of more bulky isopropyl-substituent, the tendency to phase separation is lower owing to its size.
- Application of aromatic-based PEPOs results in a more profound tendency to phase separation, which reflects the possibility of interaction between neighboring aromatic phenyl rings. Hence in this way range of hard phase dispersion is lower.
- DSC analysis showed that the PUR1 based on benzyl glycidyl ether (PBGE) has a higher glass transition temperature than PUR2 and PUR3 because of the limitation of mobility of the flexible segments.

## Figures and Tables

**Table 1:** Characterization of polyethers prepared by polymerization of different oxiranes initiated with tripotassium salt of 2,2,6,6-tetrakis(hydroxymethyl)-cyclohexanol activated 18C6 and quenched with water; [monomer]_o_ = 3.0 mol/dm^3^; [I]_o_ = 0.02 mol/dm^3^; [18C6]_o_ = 0.04 mol/dm^3^.

No.	Monomer	Polymer Fractions (%)	*M*_n_ (SEC)	M_w_/*M*_n_ (SEC)
1	BO	-	2600	1.08
2	IPGE	a(38)	5800	1.03
		b(27)	3200	1.04
		c(35)	1700	1.10
3	AGE	a(32)	4800	1.05
		b(48)	4000	1.08
		c(20)	2000	1.11
4	PGE	a(78)	6000	1.08
		b(22)	2100	1.10
5	BGE	a(25)	4000	1.05
		b(65)	2000	1.07
		c(10)	1200	1.09

**Table 2:** Hydrogen bond index for coupling of hard segments (R), range of phase separation (RPS), range of hard phase dispersion (RPD) of PURs based on PEPOs.

Name	PEPOs	CO Free ν/cm^−1^	CO Bound Non-or ν/cm^−1^	CO Bound or ν/cm^−1^	R	RPS ^(1)^ %	RPD ^(2)^ %
PUR1	PBGE	1730.0	1710.1	1704.2	7.48	88	12
PUR2	PBO	1729.4	1715.0	1703.9	2.66	73	27
		1747.0		1695.2			
				1685.0			
PUR3	PIPGE	1744.9	1717.8		2.37	70	30
		1732.1	1710.5				

^(1)^ calculated with the formula: $RPS=\left(\frac{R}{R+1}\right)\cdot 100\;\left[\%\right];\;$ ^(2)^ calculated with the formula: RDP=100−RPS[%].

**Table 3:** Thermal characteristic of cross-linked polyurethane PUR1 (PBGE and PMDI), PUR2 (PBO and PMDI), PUR3 (PIPGE and PMDI), where T_max_—temperature for maximum rate of mass loss.

Name	T5 wt%	T15 wt%	1st Decomposition Step		2nd Decomposition Step		3rd Decomposition Step		700 °C Residues
	[°C]	[°C]	Δ Mass Loss [wt%]	T_max_ [°C]	Δ Mass Loss [wt%]	T_max_ [°C]	Δ Mass Loss [wt%]	T_max_ [°C]	(%)
PUR1	338.3	378.6	-	-	74.57	403.0	-	-	16.59
PUR2	306.4	335.5	3.41	277.9	39.22	363.5	36.10	393.9	6.95
PUR3	311.3	352.1	6.35	309.5	75.70	381.9	-	-	12.34
